# McMYB4 improves temperature adaptation by regulating phenylpropanoid metabolism and hormone signaling in apple

**DOI:** 10.1038/s41438-021-00620-0

**Published:** 2021-08-01

**Authors:** Suxiao Hao, Yanfen Lu, Zhen Peng, Enying Wang, Linke Chao, Silin Zhong, Yuncong Yao

**Affiliations:** 1 Beijing Advanced Innovation Center for Tree Breeding by Molecular Design, Beijing University of Agriculture, Beijing, 102206 China; 2 Beijing Bei Nong Enterprise Management Co. Ltd, Beijing, 102206 China; 3 Plant Science and Technology College, Beijing University of Agriculture, Beijing, 102206 China; 4 Beijing Key Laboratory for Agricultural Application and New Technique, Beijing University of Agriculture, Beijing, 102206 China; 5 College of Forestry, Beijing Forestry University, Beijing, 100083 China; 6 College of Life Science, The Chinese University of Hong Kong, Hong Kong, China

**Keywords:** Transcriptional regulatory elements, Agricultural genetics

## Abstract

Temperature changes affect apple development and production. Phenylpropanoid metabolism and hormone signaling play a crucial role in regulating apple growth and development in response to temperature changes. Here, we found that *McMYB4* is induced by treatment at 28 °C and 18 °C, and *McMYB4* overexpression results in flavonol and lignin accumulation in apple leaves. Yeast one-hybrid (Y1H) assays and electrophoretic mobility shift assays (EMSAs) further revealed that McMYB4 targets the promoters of the flavonol biosynthesis genes *CHS* and *FLS* and the lignin biosynthesis genes *CAD* and *F5H*. *McMYB4* expression resulted in higher levels of flavonol and lignin biosynthesis in apple during growth at 28 °C and 18 °C than during growth at 23 °C. At 28 °C and 18 °C, McMYB4 also binds to the *AUX*/*ARF* and *BRI*/*BIN* promoters to activate gene expression, resulting in acceleration of the auxin and brassinolide signaling pathways. Taken together, our results demonstrate that McMYB4 promotes flavonol biosynthesis and brassinolide signaling, which decreases ROS contents to improve plant resistance and promotes lignin biosynthesis and auxin signaling to regulate plant growth. This study suggests that McMYB4 participates in the abiotic resistance and growth of apple in response to temperature changes by regulating phenylpropanoid metabolism and hormone signaling.

## Introduction

As an important environmental factor, temperature often impacts plant growth, development and productivity during the growing season^1^. Therefore, increasing attention has been directed toward the regulation of plants in response to temperature changes^2^. Plants are believed to activate protective mechanisms during their adaptation to temperature changes by balancing resistance and growth. For example, under high-temperature conditions, SLG1 (Slender Guy 1) could encode cytosolic tRNA 2-thiolation protein 2 (RCTU2) by regulating its promoter and coding regions, which led to an increase in thiolated tRNA and enhanced resistance and thus improved rice yields^3^. In *Arabidopsis thaliana*, the photosystem II (PSII)-associated protein PSB27 downregulated PSII electron transport under low-temperature conditions to affect plant photosynthesis and growth^4^.

Apple *(Malus domestica)* is frequently exposed to challenges from various environmental factors. Temperature has a serious impact on growth and development processes, which affects apple yields^5^. At a low temperature (15 °C), McMYB10 bound to the promoter of flavonoid 3’-hydroxylase (*McF3’H*) and promoted its expression to enhance anthocyanin accumulation in crabapple leaves and calli, which promoted apple coloration^6^. In apple, MdERF1B-MdCIbHLH1 upregulated the expression of the ethylene biosynthesis genes *MdACO1* and *MdERF3* and promoted ethylene production, which positively modulated the response of apple to 4 °C^7^. Therefore, substance metabolism and hormone signaling in apple plants play a crucial role in their response to temperature changes.

Phenylpropanoid metabolism is an important secondary metabolic process in the plant kingdom^8^. Flavonol, a key component in this metabolic pathway, can increase plant resistance and regulate plant growth. For instance, the flavonol contents in leaves of a hybrid *Arabidopsis* line are higher than those in leaves of Columbia plants, and this difference is accompanied by greater accumulation of soluble sugars and a higher proline content after a 14-day low-temperature treatment, which increases plant freezing tolerance^9^. Moreover, the flavonol contents in *Arabidopsis thaliana* were also correlated with a shortened plant stature. The flavonoid 3-O-glucosyltransferase mutant *ugt78d2* regulated the flavonol glycoside pattern and reduced polar auxin transport (PAT) in shoots, which resulted in a dwarf-type stature^10^. Lignin, as a complex phenolic polymer, can affect the cell wall and vascular structure and thereby regulate plant growth and resistance^11,12^. For example, expression of the p-coumaroyl ester 3-hydroxylase gene *C3’H* promoted lignin biosynthesis and changed the cell wall structure, thereby regulating plant growth and dwarfing in rice^13^. In maize roots, the fraction of the lignocellulosic complex in the cell wall was significantly increased, which resulted in lignin deposition and root growth promotion^14^. Furthermore, chilling temperatures (8 °C/4 °C) also promoted lignified xylem deposition in cell walls by regulating the transcriptional levels of secondary cell wall (SCW) genes in *Eucalyptus gunnii* × *Eucalyptus dalrympleana* hybrids. The expression levels of most genes involved in lignin biosynthesis were upregulated and contributed to the cold tolerance of the hybrids^15^. These results indicate that flavonol and lignin play an important role in plant growth and resistance. However, their effects on apple in response to temperature changes need further study.

Phytohormone signaling can also regulate plant growth and resistance in response to temperature changes. Under low-temperature conditions, RNA sequencing (RNA-seq) revealed that the C-repeat binding transcription factor (CBF1) promoted the expression of genes involved in auxin, GA, and cytokinin signaling in peach and affected hormone homeostasis and growth^16^. In apple, MdPIF4 could transactivate the *MdYUCCA8a* promoter, which promoted indole-3-acetic acid (IAA) accumulation and further improved plant height, thus affecting apical dominance and silique malformation under high-temperature conditions^17^. Moreover, high temperatures also promoted the expression of auxin biosynthesis pathway genes (*GmYUCCA3*, *GmYUCCA5* and *GmYUCCA7*) to increase IAA accumulation and promoted soybean hypocotyl elongation^18^. However, under freezing conditions, overexpression of *BYPASS1-LIKE (B1L)* decreased hypocotyl length and fresh weight in *Arabidopsis* by regulating the BR signaling pathway. Furthermore, the *TRANSTHYRETIN-LIKE (TTL) ttl-1* mutant promoted freezing tolerance. Therefore, *B1L* overexpression promoted plant freezing tolerance by interacting with TTL^19^. BR could also positively regulate the net photosynthetic rate and stomatal conductance under low-temperature treatment (8 °C), which promoted the growth and development of tung trees^20^. These results suggest that phytohormones have a major impact on the regulation of plant growth and resistance in response to temperature changes.

MYB transcription factors act as key regulators and affect plant growth and resistance by regulating target genes related to substance synthesis and metabolism^21^. More recent studies in *Arabidopsis* have shown that overexpression of *AtHY5* and *AtMYB12* enhanced flavonol accumulation under low-temperature conditions and promoted plant resistance to freezing conditions^22^. In apple plants, MdMYB308L could interact with MdbHLH33 and undergo MdMIEL1-mediated protein degradation to regulate cold tolerance^23^. In maize, sorghum and rice, MYB31 and MYB42 also promoted the expression of the key genes *4CL2*, *F5H*, and *CSE* in the lignin biosynthesis pathway, thus altering lignin production and promoting plant growth^24^. The R2R3-MYB transcription factor MYB15 could accelerate lignification and thereby affect plant growth and resistance in energy-related and agricultural crops by regulating MYB-responsive elements in genes involved in secondary wall biosynthesis^25^. Therefore, investigating the involvement of MYB transcription factors in the response to temperature changes by regulating substance synthesis in apple would be interesting.

In this study, we overexpressed *McMYB4* in *Malus domestica* “Golden Delicious” apple tissue culture seedlings. The results showed that flavonol and lignin accumulation in *McMYB4*-overexpression (OE) lines is correlated with high *McMYB4* expression under different temperature conditions. Y1H assays and EMSAs revealed that McMYB4 not only targets the promoters of the flavonol biosynthesis genes *CHS* and *FLS* but also binds to the promoters of the lignin biosynthesis genes *CAD* and *F5H* to promote more flavonol and lignin biosynthesis at 28 °C and 18 °C than at 23 °C. McMYB4 also promotes IAA and BR signaling pathways by upregulating the expression of *AUX/ARF* and *BRI/BIN*. Our findings suggest that McMYB4 improves the temperature adaptation of apple by regulating phenylpropanoid metabolism and hormone signaling.

## Results

### *McMYB4* cloning and temperature response analysis

**Fig. 1:**
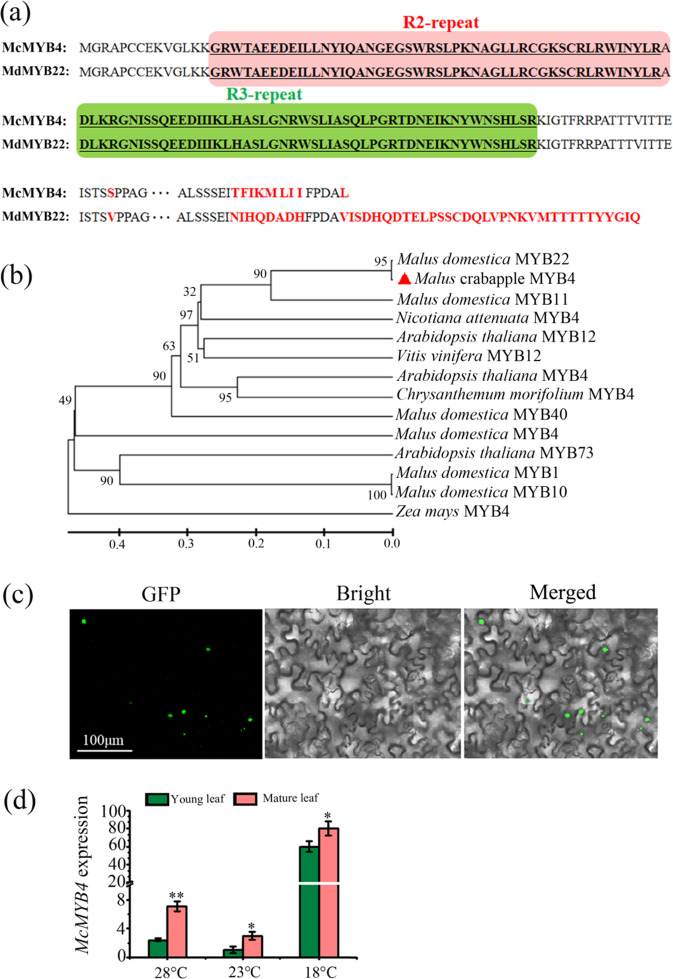
Bioinformatics and expression analysis of *McMYB4*. **a** Amino acid sequence analysis of McMYB4. **b** Phylogenetic analysis of McMYB4. **c** Subcellular localization of McMYB4. Bar = 100 μm. **d** Expression analysis of *McMYB4*. Each bar indicates the mean ± SD of three repeated experiments (*P < 0.05, **P < 0.01, Student’s *t* test)

The novel gene *McMYB4*, which was identified in the *Malus* crabapple cultivar “Royalty”, contains conserved R2 and R3 DNA-binding domains, and a 33 amino acid deletion was found in the C-terminus of McMYB4 compared with that of *M. domestica* MdMYB22 (Fig. 1a). The phylogenetic tree showed that the similarity between McMYB4 and MdMYB22 was 95% (Fig. 1b), and a subcellular localization analysis revealed that McMYB4 is located in the nucleus (Fig. 1c).

*MdMYB22* was significantly induced in the “Gala” apple cultivar by low temperature exposure, and its expression also correlated strongly with anthocyanin accumulation^21^. To explore the temperature response of McMYB4 in “Royalty”, we cultivated tissue culture seedlings of “Royalty” under 28 °C and 18 °C conditions with 23 °C as the control. qRT-PCR indicated that 28 °C treatment increased *McMYB4* expression by 2.4-fold in young leaves and 2.1-fold in mature leaves. In addition, 18 °C treatment increased *McMYB4* expression by 60-fold in young leaves and 21-fold in mature leaves (Fig. 1d). Therefore, *McMYB4* expression is responsive to temperature changes in *Malus* crabapple.

### McMYB4 promotes flavonol and lignin biosynthesis in response to temperature changes

**Fig. 2:**
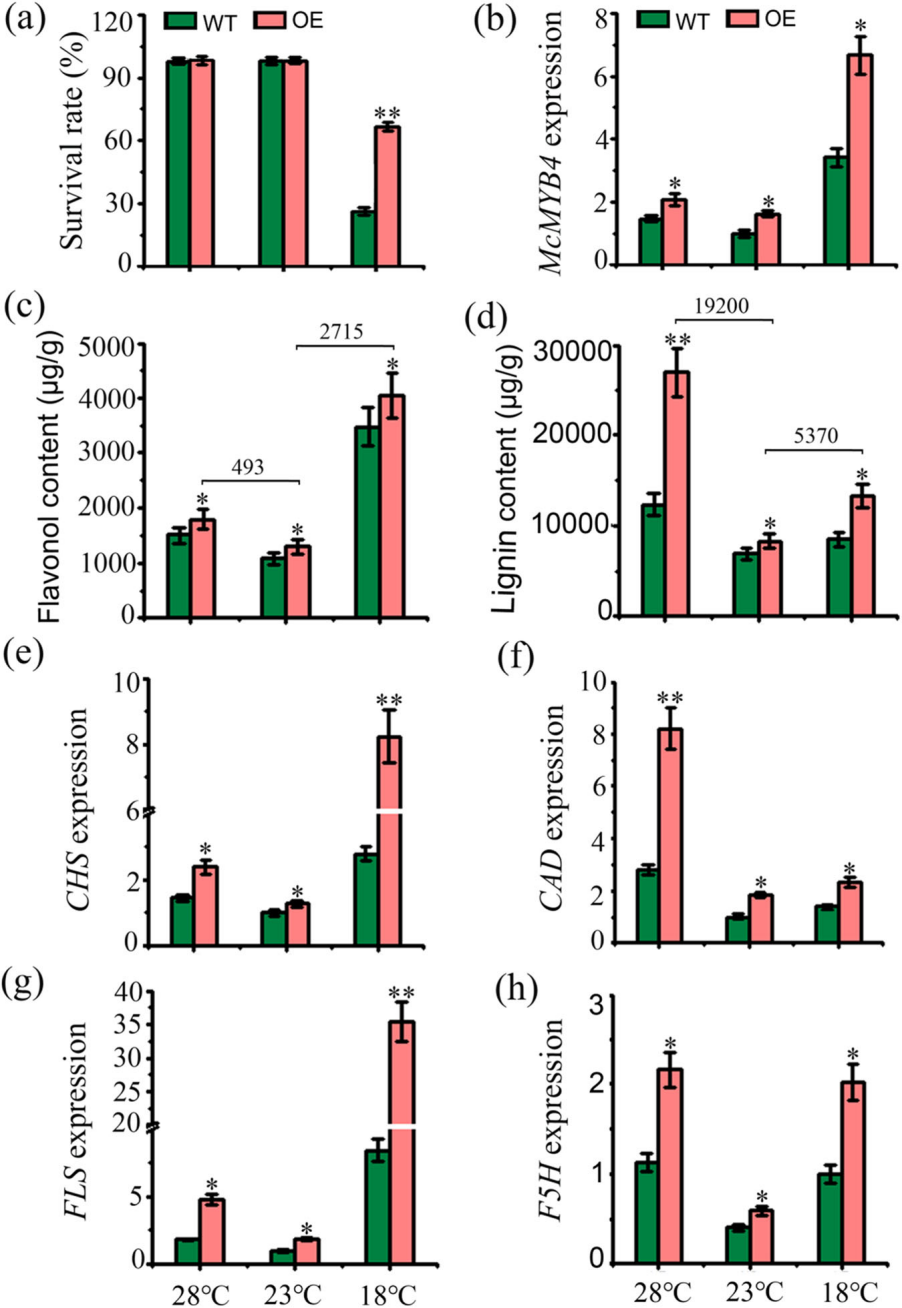
Flavonol and lignin biosynthesis in the WT and *McMYB4*-OE lines in response to temperature changes. **a** Survival rate of the WT and OE lines during treatment at 28 °C and 18 °C; 23 °C served as the control. **b** Expression levels of *McMYB4* in the WT and OE lines treated at 28 °C and 18 °C; 23 °C served as the control. **c**, **d** Flavonol and lignin contents in the WT and OE lines grown at 28 °C and 18 °C; 23 °C served as the control. **e**–**h** Expression levels of structural genes in the flavonol and lignin biosynthesis pathways (*CHS*, *CAD*, *FLS*, and *F5H*) in the WT and OE lines treated at 28 °C and 18 °C; 23 °C served as the control. Each bar indicates the mean ± SD of three repeated experiments (*P < 0.05, **P < 0.01, Student’s *t* test)

To further explore the temperature response of McMYB4 in “Golden Delicious” apple, we transformed the *McMYB4* overexpression vector into “Golden Delicious” apple tissue culture seedlings. We selected higher-expressing *McMYB4*-OE transgenic plants (lines 1, 10, and 12) and “Golden Delicious” apple tissue culture seedlings (used as the WT control) and grew them at 28 °C, 18 °C, and 23 °C (with 23 °C as the control) for seven days. The results showed that the WT lines wilted, and *McMYB4* overexpression alleviated the severity of wilting (S-Fig. 2). At 18 °C, the survival rate of the WT lines was 27%, and that of the *McMYB4*-OE lines was 67%. The survival rate of the WT and *McMYB4*-OE lines was ~100% at 28 °C (Fig. 2a). qRT-PCR analysis indicated that *McMYB4* expression in the *McMYB4*-OE lines was 1.3-fold and 4.1-fold higher at 28 °C and 18 °C than at 23 °C, respectively (Fig. 2b). *McMYB4* overexpression promoted flavonol and lignin accumulation. HPLC results showed that the flavonol contents in the *McMYB4*-OE lines grown at 28 °C and 18 °C were 493 μg/g and 2715 μg/g higher than those at 23 °C, respectively, which were accompanied by upregulated *CHS* and *FLS* expression (Fig. 2c, e, g). The lignin content in the *McMYB4*-OE lines grown at 28 °C and 18 °C was increased by 19200 μg/g and 5370 μg/g, respectively, compared with that in the *McMYB4*-OE lines grown at 23 °C, and these increases were accompanied by upregulated *CAD* and *F5H* expression (Fig. 2d, f, h). Taken together, the results indicate that *McMYB4* overexpression enhances the temperature adaptation of apple plants by promoting flavonol and lignin biosynthesis at 28 °C and 18 °C.

### Transcriptome sequencing analysis of *McMYB4* transgenic “Golden Delicious” apple

**Fig. 3:**
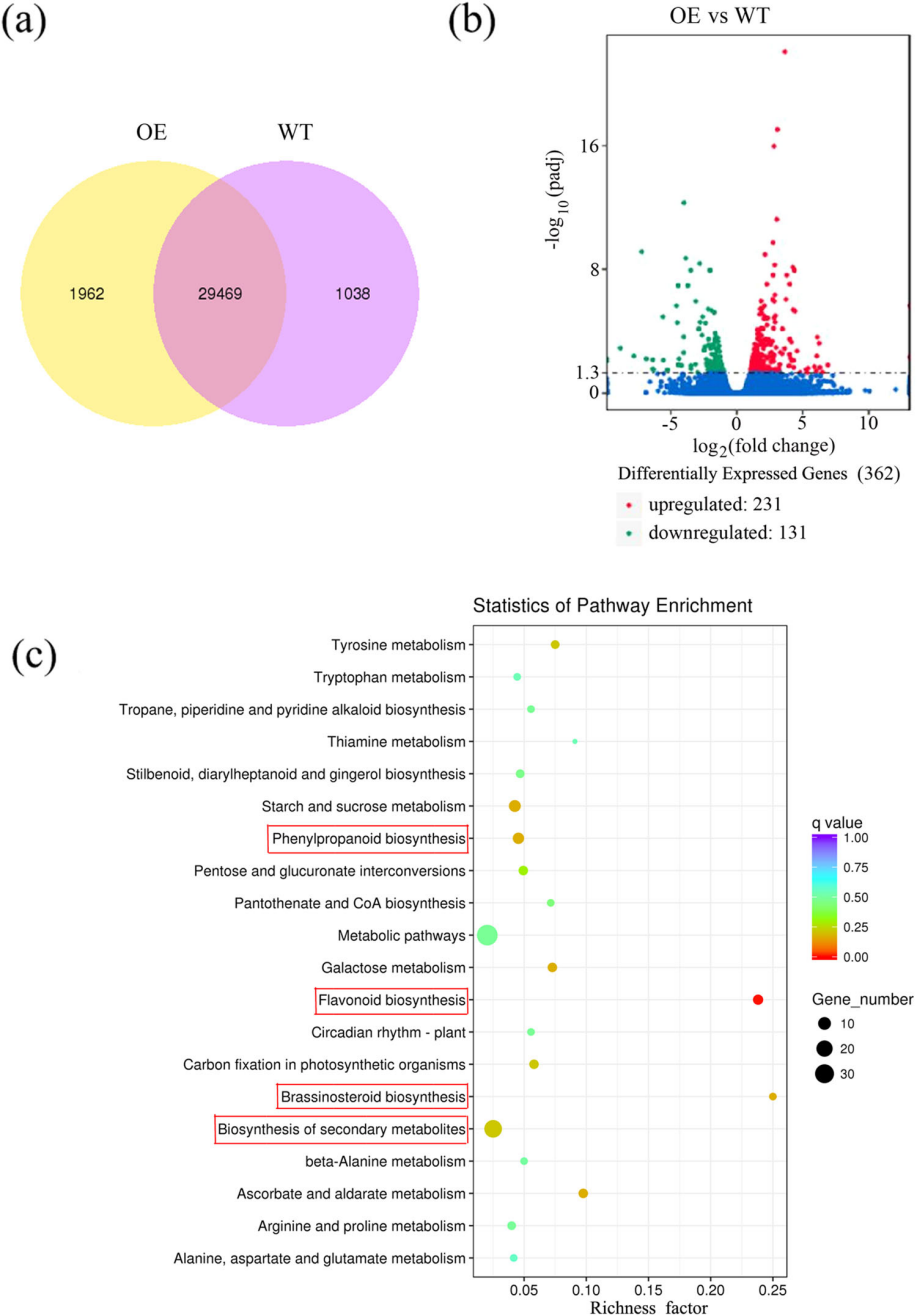
Transcriptome analysis. **a** Venn plot analysis of the WT and *McMYB4*-OE lines. **b** DEG volcano plot analysis of the WT and *McMYB4*-OE lines. Red indicates upregulation, and green represents downregulation. **c** KEGG enrichment analysis. The ordinate indicates the pathway name, the abscissa shows the richness factor, the size of the points represents the number of DEGs in the pathway, and the color of the points corresponds to different *q* value ranges

We selected *McMYB4*-OE plants (line 1) and a WT line for transcriptome sequencing analysis. As shown in the Venn plot, 1962 genes were specifically expressed in the *McMYB4*-OE line, 1038 genes were specifically expressed in the WT line, and 29469 genes were expressed in both the *McMYB4*-OE and WT lines (Fig. 3a). Differentially expressed gene (DEG) analysis of the *McMYB4*-OE and WT lines revealed 231 upregulated genes and 131 downregulated genes based on the criteria |log2(Foldchange)| > 1 and a *q* < 0.005 (Fig. 3b). KEGG analysis indicated that the relevant regulatory pathways of the DEGs were mainly associated with secondary metabolite processes, such as phenylpropanoid metabolism, flavonoid biosynthesis, and brassinosteroid biosynthesis pathways (Fig. 3c). Moreover, diphenyl boric acid 2-aminoethyl ester (DPBA) staining also revealed that the *McMYB4*-OE line contained higher flavonol contents. Microscopy observations of cross-sections of the petioles under UV light revealed that the *McMYB4*-OE line contained a higher lignin content and more vascular tissue than the WT line (S-Fig. 1c).

### McMYB4 binds to the promoters of *CHS*, *FLS*, *CAD*, and *F5H* involved in flavonol and lignin biosynthesis

**Fig. 4:**
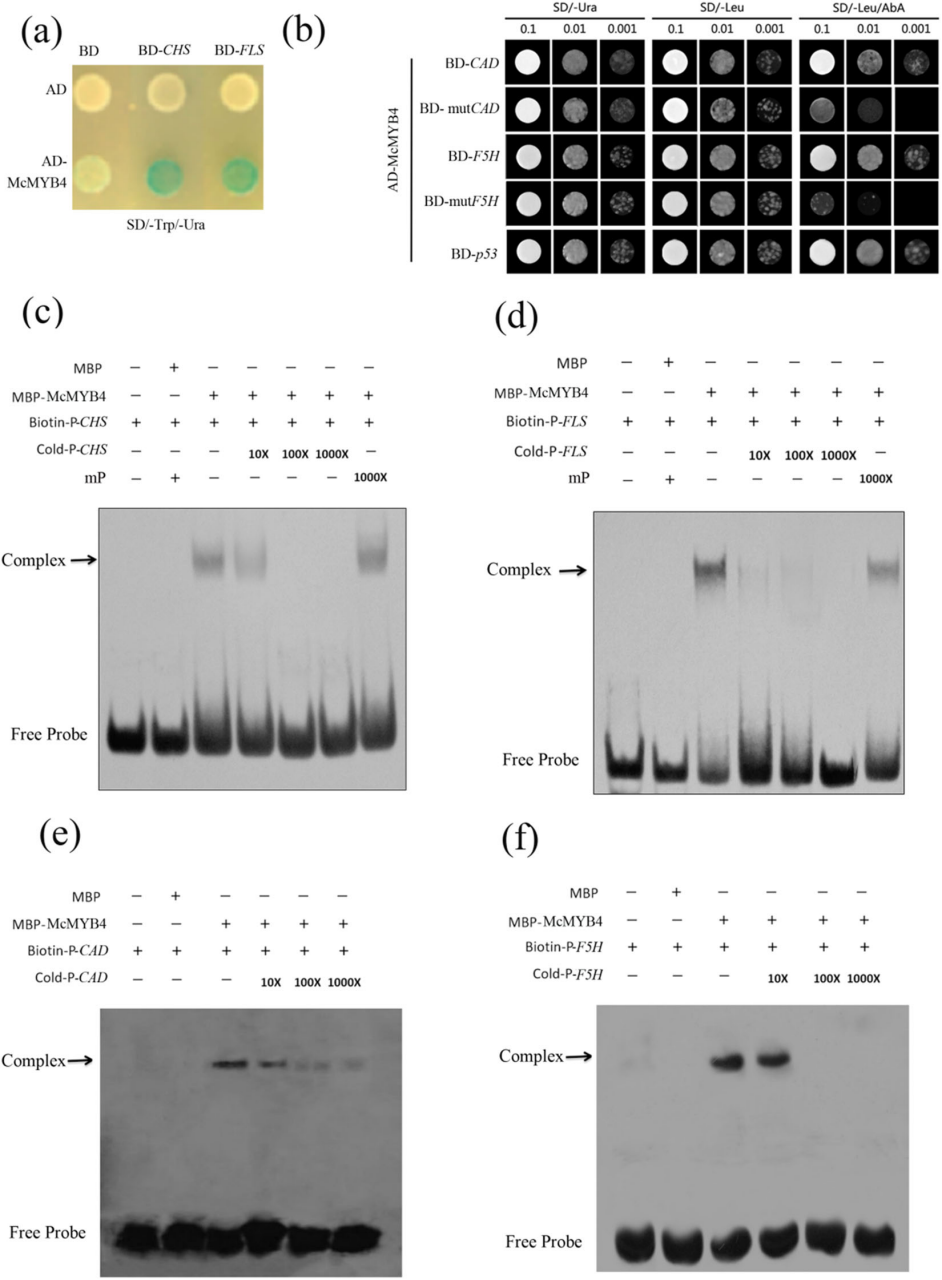
Analysis of the target genes of McMYB4 in the phenylpropane metabolism pathway. **a** Binding of McMYB4 to the promoters of the flavonol biosynthesis genes *CHS* and *FLS* as measured by Y1H assays. **b** Binding of McMYB4 to the promoters of the lignin biosynthesis genes *CAD* and *F5H* as determined by Y1H assays. **c**, **d** Binding of McMYB4 to the promoters of the flavonol biosynthesis genes *CHS* and *FLS* as measured by EMSAs. mP: mutant probe. **e**, **f** Binding of McMYB4 to the promoters of the lignin biosynthesis genes *CAD* and *F5H* as determined by EMSAs

To further verify the target genes of McMYB4 in the flavonol biosynthesis pathway, we performed Y1H assays. The results revealed that the *LacZ* reporter gene was activated in yeast transformants containing the AD*-*McMYB4 vector and vectors harboring the promoters of the target genes BD*-CHS* and BD*-FLS* (Fig. 4a). EMSA results also showed that McMYB4 binds to the biotin-labeled promoters of *CHS* and *FLS* (Fig. 4c, e). Therefore, these results indicate that McMYB4 can bind to the promoters of *CHS* and *FLS* to promote flavonol biosynthesis.

Y1H assays also showed that the *AbAi* reporter gene was activated in yeast transformants containing the AD*-*McMYB4 vector and vectors harboring the promoters of the target genes *CAD* and *F5H* (Fig. 4b), and EMSAs demonstrated that McMYB4 binds to the biotin-labeled promoters of *CAD* and *F5H* (Fig. 4d, f). Therefore, these results show that McMYB4 can also bind to the promoters of *CAD* and *F5H* to promote lignin biosynthesis.

### *McMYB4* transgenic “Golden Delicious” apple responds to temperature changes by regulating IAA and BR signaling

**Fig. 5:**
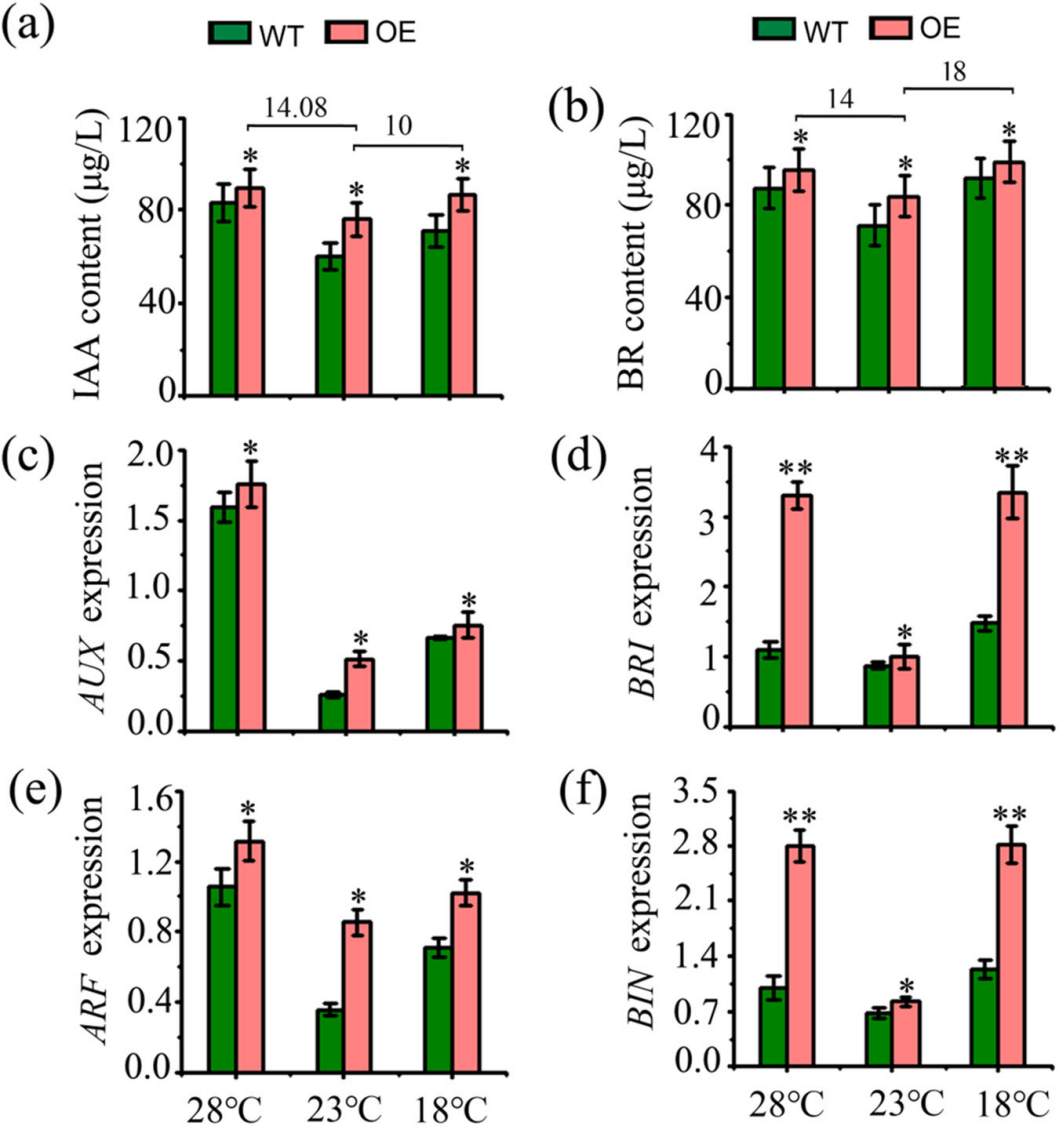
Analysis of IAA and BR signaling pathways in the WT and *McMYB4*-OE lines in response to temperature changes. **a**, **b** IAA and BR contents in the WT and OE lines grown at 28 °C and 18 °C; 23 °C served as the control. **c**, **e** Expression levels of genes in the IAA signaling pathway (*AUX* and *ARF*) in the plants subjected to the 28 °C and 18 °C treatments; 23 °C served as the control. **d**, **f** Expression levels of genes in the BR signaling pathway (*BRI* and *BIN*) with treatment at 28 °C and 18 °C; 23 °C served as the control. Each bar indicates the mean ± SD of three repeated experiments (*P < 0.05, **P < 0.01, Student’s *t* test)

**Fig. 6:**
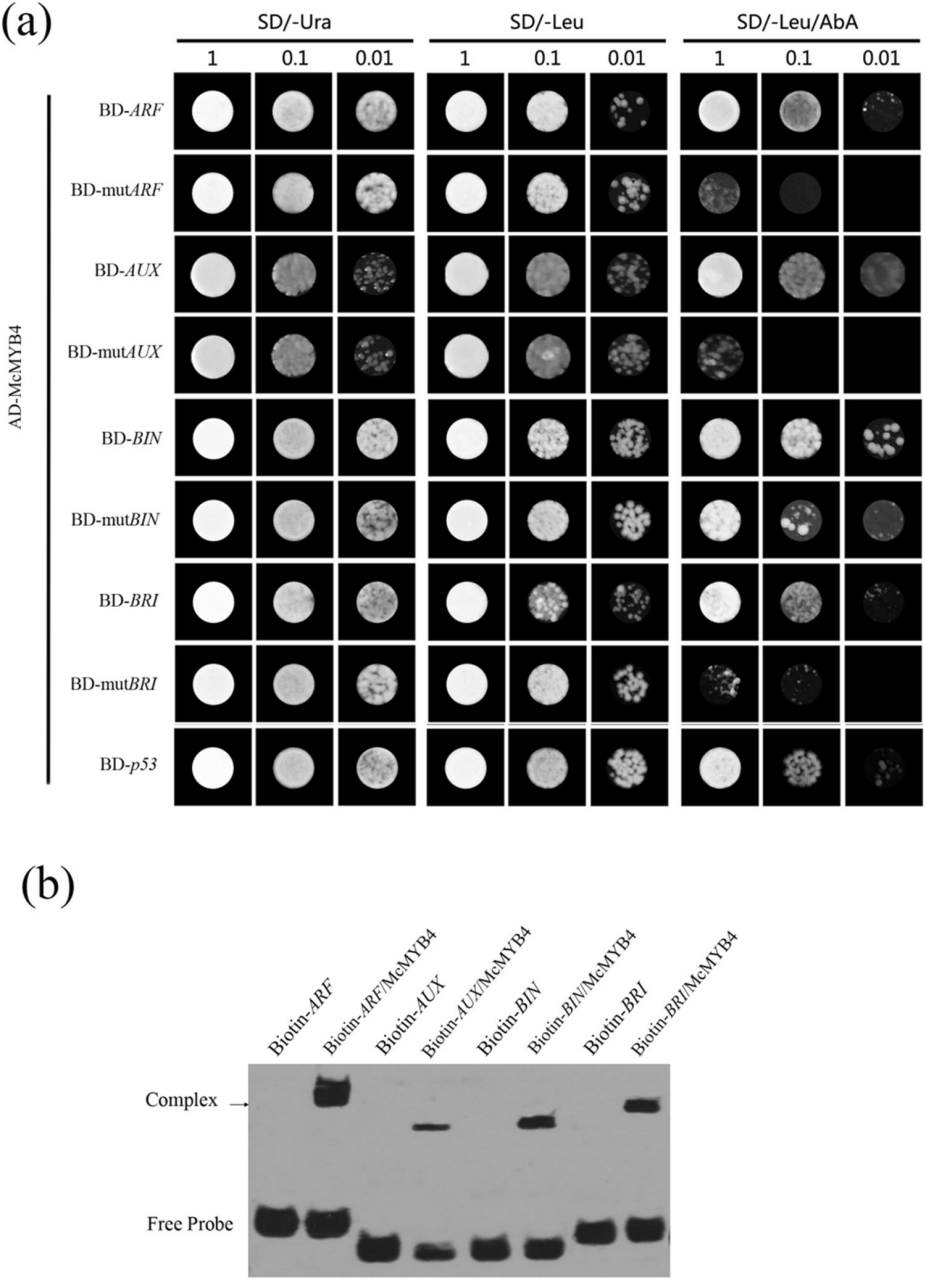
Analysis of the target genes of McMYB4 in the IAA and BR signaling pathways. **a** Binding of McMYB4 to the promoters of the IAA signaling pathway genes *ARF* and *AUX* and the BR signaling pathway genes *BIN* and *BRI* as determined by Y1H assays. **b** Binding of McMYB4 to the promoters of the IAA signaling pathway genes *ARF* and *AUX* and the BR signaling pathway genes *BIN* and *BRI* as determined by EMSAs

To analyze the function of McMYB4 in phytohormone signaling pathways in response to temperature changes, we subsequently evaluated the hormone contents of IAA and BR in the WT and *McMYB4*-OE lines. The results showed that *McMYB4* overexpression promotes IAA and BR accumulation. Compared with that at 23 °C (which served as the control), the IAA content was increased by 14.08 μg/L and 10 μg/L in the *McMYB4*-OE lines grown at 28 °C and 18 °C, respectively, and this increase was accompanied by upregulated *AUX* and *ARF* expression (Fig. 5a, c, e). In addition, the BR content was increased by 14 μg/L and 18 μg/L in the *McMYB4*-OE lines grown at 28 °C and 18 °C, respectively, compared with that at 23 °C, and this change was accompanied by upregulated *BRI* and *BIN* expression (Fig. 5b, d, f). Moreover, Y1H assays and EMSAs indicated that McMYB4 mainly activates the promoters of *AUX*, *ARF*, *BRI*, and *BIN* and promotes the IAA and BR signaling pathways (Fig. 6). Consequently, our results suggest that *McMYB4* overexpression may enhance IAA and BR accumulation by promoting the expression of genes related to the IAA and BR signaling pathways in response to 28 °C and 18 °C treatments.

### *McMYB4* overexpression decreases ROS levels in apple plants under different temperature conditions

ROS are important signaling molecules in plants that play roles in the response to temperature changes. Therefore, we analyzed ROS levels in the WT and *McMYB4*-OE lines. The leaves of the WT and *McMYB4*-OE lines at 28 °C, 23 °C, and 18 °C were stained with nitro blue tetrazolium (NBT) (dark blue) and diaminobenzidine (DAB) (dark brown). The results showed that the 28 °C and 18 °C treatments increased the intensity of DAB and NBT staining compared with that observed in the 23 °C treatment (control), and this increased intensity reflects an increase in O_2_^-^ and H_2_O_2_ accumulation. Compared with those of the WT lines, the leaves of the *McMYB4*-OE lines exhibited lower DAB and NBT staining intensities, which reflected lower levels of O_2_^-^ and H_2_O_2_ accumulation (S-Fig. 3a, b). Therefore, in the *McMYB4*-OE lines, the 28 °C and 18 °C treatments increased the O_2_^-^ content by 37 μmol/g and 18 μmol/g, respectively (S-Fig. 3c), and increased the H_2_O_2_ content by 0.62 μmol/g and 0.29 μmol/g, respectively, compared with the levels found with the control treatment (23 °C; S-Fig. 3d). The activities of APX, CAT, and SOD were also higher in the lines grown at 28 °C and 18 °C than in those grown at 23 °C as the control, and the activities of these enzymes in the *McMYB4*-OE lines were higher than those in the WT lines under the different temperature conditions (S-Fig. 3e–g). These results indicate that *McMYB4* overexpression decreases ROS levels in apple plants by increasing APX, CAT, and SOD activities and thus increases the resistance of plants to 28 °C and 18 °C.

## Discussion

Temperature changes can regulate apple growth and influence apple production and quality. For example, silencing of the MADS-box gene affected the dormancy and bud break of apple trees in response to chilling temperatures, resulting in an ever-growing or evergreen phenotype and decreased fruit production^26^. In “Fuji” apple, the expression of a B-box transcription factor, MdCOL4, was promoted by high temperatures. MdCOL4 downregulated *MdMYB1* expression by interacting with MdHY5 under high-temperature conditions, resulting in decreases in apple color intensity and growth^27^. Therefore, understanding the response mechanisms for adapting to temperature changes is crucial for improving apple yields.

### McMYB4 promotes flavonol and lignin accumulation in apple plants adapting to temperature changes

Plants can activate a temperature response by regulating phenylpropanoid metabolism^28^. In tea plants, low-temperature treatment induced high *CsHCT* transcription, resulting in the production of chlorogenic acid or acylated flavonol glycosides^29^. In mulberry leaves, low temperatures could also promote the expression of the *UFGT* gene and lead to flavonoid accumulation^30^. However, the function of McMYB4 in flavonol biosynthesis pathways in response to temperature changes is unclear. In our study, the expression of *CHS* and *FLS*, which have been verified as target genes of McMYB4, was increased in the *McMYB4*-OE transgenic lines at 28 °C and 18 °C (Figs. 2, 4). In addition, MYB TFs also regulate lignin biosynthesis in plant growth and development processes^31^. In loquat fruit, EjAP2-1 interacted with EjMYB1 and EjMYB2 and transrepressed the promoter of the lignin biosynthesis gene *Ej4CL1* to regulate postharvest lignification during low-temperature storage^32^. In maize, ZmMYB31 downregulated the expression of genes involved in monolignol biosynthesis to significantly reduce the lignin content and increase cell wall degradability^33^. In addition, PvMYB4 acts as a master repressor of lignin biosynthesis by decreasing the lignin content and S/G lignin ratio in switchgrass plants^34^. However, MdUGT88F1-mediated phloridzin biosynthesis could increase the levels of lignin and cell wall polysaccharides in apple trees, resulting in increases in internode length and stem and adventitious root numbers and improved growth^35^. In our study, the lignin content was increased in the *McMYB4*-OE lines grown at 28 °C and 18 °C compared with the plants grown at 23 °C, and this increase was accompanied by upregulated *CAD* and *F5H* expression (Figs. 2, 4). Therefore, McMYB4 coordinates the temperature response in apple plants by promoting flavonol and lignin biosynthesis at 18 °C and 28 °C.

### The response of McMYB4 to temperature changes is related to the IAA and BR signaling pathways in apple plants

Phytohormone signaling can regulate plant resistance and growth in response to temperature changes^36^. In *Arabidopsis thaliana*, the chromatin-modifying enzyme HDA9 mediated histone deacetylation of a rate-limiting enzyme involved in auxin biosynthesis under high-temperature conditions and promoted auxin accumulation and thermomorphogenesis^37^. Under 28 °C conditions, the precursor of bioactive GA12 promoted the degradation of DELLAs and induced PIF4-induced upregulation of *IAA19/29* expression and hypocotyl elongation in *Arabidopsis*^38^. Similarly, under 28 °C conditions, COP1 could regulate BZR in the BR signaling pathway and promote hypocotyl elongation and petiole growth in *Arabidopsis*^39^. Under low-temperature conditions, RNA-sequencing analysis showed that BR treatment upregulated the expression of several key genes involved in chlorophyll biosynthesis, promoting the photosynthetic capacity and growth of wucai^40^. Moreover, *Arabidopsis thaliana SCD1* (stomatal cytokinesis defective 1), a temperature-sensitive allele, could regulate auxin transport and auxin-induced gene expression and affect the gravitropic growth of *Arabidopsis* in response to different temperatures (25 °C and 18 °C) by regulating PIN protein trafficking^41^. In our study, *McMYB4* overexpression increased not only the IAA and BR contents but also the transcript levels of *AUX*/*ARF* and *BRI/BIN* in the IAA and BR signaling pathways (Figs. 5, 6), suggesting that the response of McMYB4 to temperature changes is related to the IAA and BR signaling pathways in apple plants.

### McMYB4 might participate in abiotic resistance and growth by promoting phenylpropanoid biosynthesis and phytohormone signaling in response to temperature changes

Phenylpropanoids are a large class of secondary metabolites involved in plant resistance and growth in coordination with phytohormone signaling^42^. For example, *p35S:F3H* transgenes increased flavonol levels, and flavonol accumulation reduced ROS accumulation, promoting antioxidant activity in tomato under high-temperature conditions^43,44^. In addition, cytokinin could also induce flavonol accumulation in the root transition zone, and a high flavonol content decreased the superoxide radical content, promoting root resistance in *Arabidopsis*^45^. Moreover, in red mango fruit, RNA-seq analysis suggested that the genes involved in the brassinosteroid signaling pathway were upregulated and increased plant resistance to light^46^. In addition, in brassinosteroid signaling, SERK2, as a BR signaling component, could interact with OsBRI1 to promote early BR signaling and enhance grain size and salt tolerance^47^. Therefore, flavonol biosynthesis and BR signaling have important functions in plant resistance, and during this process, ROS act as plant signals to respond to environmental changes^48^^.^ In watermelon, the H_2_O_2_ content increased and improved adaptation to 4 °C treatment^49^. In our study, the flavonol levels in the *McMYB4*-OE lines grown at 28 °C and 18 °C were increased by 493 μg/g and 2715 μg/g, respectively, compared with those in the control treatment, and the BR contents were increased by 14 μg/L and 18 μg/L in these lines, respectively. The increases in the contents of these metabolites facilitate scavenging of the ROS induced by temperature changes (S-Fig. 3).

As an important component of the cell wall, lignin can affect plant growth. In tea plants, melatonin treatment modified the expression of enzyme genes in the lignin synthesis pathway, increased the lignin content, and promoted plant growth and development^50^. Similarly, in plant growth and development processes, auxin can also affect cell division and elongation to regulate plant growth. For instance, in *Arabidopsis*, the auxin transcriptional repressor IAA3 interacted with light-controlled PIF transcription factors to regulate hypocotyl growth, and disruption of IAA3 led to an elongated hypocotyl under different light intensity conditions^51^. In our study, McMYB4 also promoted lignin biosynthesis and IAA signaling at 28 °C and 18 °C. The lignin content in the *McMYB4*-OE lines grown at 28 °C and 18 °C was increased by 19200 μg/g and 5370 μg/g, respectively, compared with that found in the plants treated at 23 °C, and the IAA content was increased by 14.08 μg/L and 10 μg/L in these lines, respectively. Taken together, the results demonstrated that McMYB4 upregulates flavonol biosynthesis and brassinolide signaling to improve plant resistance during growth at 18 °C. Moreover, McMYB4 upregulates lignin biosynthesis and auxin signaling and thereby promotes plant growth at 28 °C.

**Fig. 7:**
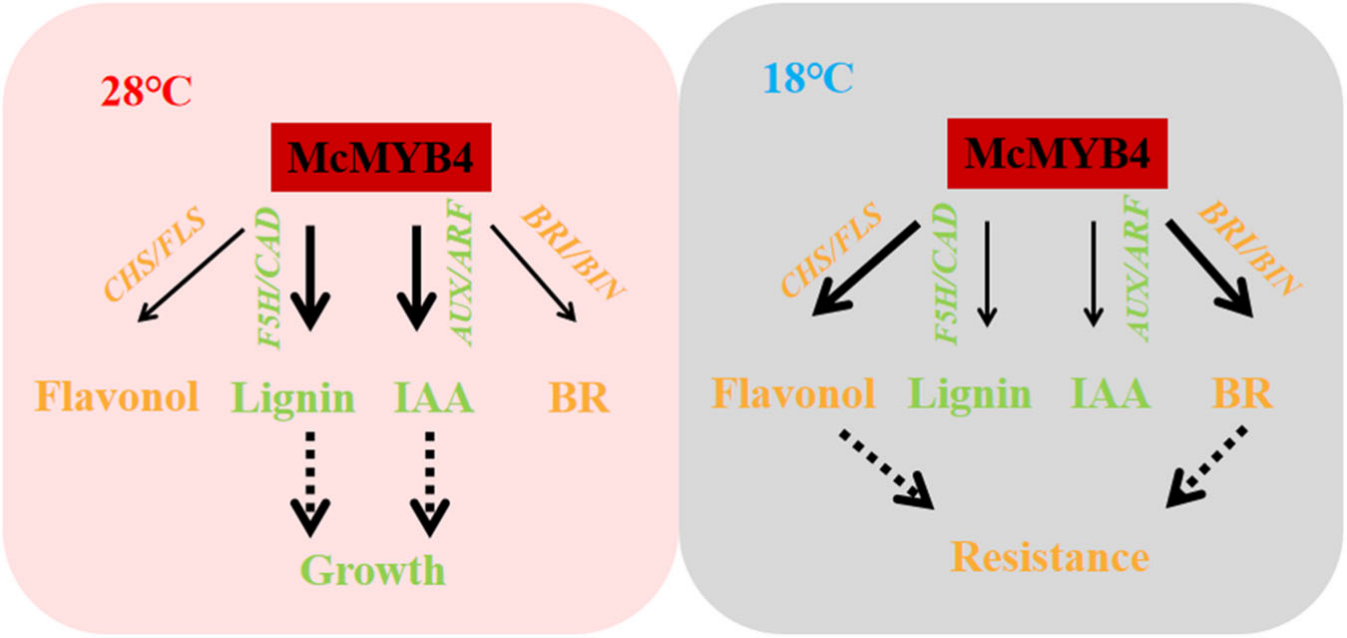
The regulation model of McMYB4 under different temperature conditions. Yellow: flavonol metabolism and BR signaling pathways; Green: lignin metabolism and IAA signaling pathways; Coarse arrow: strong promotion; Thin arrow: weak promotion; Dotted arrow: proposed McMYB4 regulated phenylpropane metabolism and hormone signaling to balance growth and resistance homeostasis.

In conclusion, McMYB4 increases flavonol and lignin accumulation by promoting the expression of *CHS/FLS* and *F5H/CAD* in apple at 18 °C and 28 °C. Moreover, McMYB4 promotes *AUX*/*ARF* and *BRI/BIN* expression and is associated with IAA and BR signaling. Therefore, McMYB4 plays a role in plant resistance and growth by regulating phenylpropanoid metabolism and hormone signaling in response to temperature changes in apple (Fig. 7).

## Materials and methods

### Plant materials and growth conditions

Tissue culture seedlings of the *Malus* crabapple cultivar “Royalty”, *M. domestica* “Golden Delicious” apple (WT), and transgenic “Golden Delicious” apple (lines 1, 10, and 12) were cultivated at the Tissue Culture Center of Beijing University of Agriculture under a 16/8-h photoperiod (1800–2000 lx) and 70% humidity at 23 °C for one month. Subsequently, 15 strains were selected from line 1, line 10 and line 12. Five of 15 strains of the “Royalty” plants, five of 15 strains of the WT plants and five of 15 strains of the *McMYB4*-OE transgenic plants (lines 1, 10, and 12) were grown in an illumination incubator under a 16/8-h photoperiod (1800–2000 lx) and 70% humidity at 23 °C as the control. Five of 15 strains of the “Royalty” plants, five of 15 strains of the WT plants and five of 15 strains of the *McMYB4*-OE transgenic plants (lines 1, 10, and 12) were grown in an illumination incubator under a 16/8-h photoperiod (1800–2000 lx) and 70% humidity at 28 °C (high day temperature). Five of 15 strains of the “Royalty” plants, five of 15 strains of the WT plants and five of 15 strains of the *McMYB4*-OE transgenic plants (lines 1, 10, and 12) were grown in an illumination incubator under a 16/8-h photoperiod (1800–2000 lx) and 70% humidity at 18 °C (low night temperature). After seven days of treatment^52^, all the samples were frozen in liquid nitrogen and stored at −80 °C for follow-up assays.

### qRT-PCR analysis

Total RNA was extracted from the leaves in liquid nitrogen under RNase-free conditions via guanidine thiocyanate solution. qRT-PCR was then performed using SYBR^®^ Premix Ex Taq^TM^ II (Perfect Real Time) (Takara) and a CFX96^TM^ Real Time System (Bio-Rad, USA). *Malus 18* *S* was used as an internal control, and expression differences were calculated according to the 2^(−ΔΔCt)^ analysis method. The specific primers used for the qRT-PCR analysis are listed in Supplementary Table 1.

### Ectopic expression of *McMYB4* in “Golden Delicious” apple seedlings

We cloned *McMYB4* from the *Malus* crabapple cultivar “Royalty”, constructed a *pH7FWG2-McMYB4-GFP*-overexpression vector containing XbaI and SacI sites and transformed it into *Agrobacterium tumefaciens* GV3101. After the *A. tumefaciens* populations grew to saturation (OD_600_ = 0.8) in Luria-Bertani media, the culture was centrifuged. The thallus was resuspended in a solution of 10 mM MgCl_2_, 10 mM 2-(N-morpholino) ethanesulfonic acid (MES), and 150 mM acetosyringone and maintained at room temperature for 2 h. The tissue culture seedlings of “Golden Delicious” apple were infected with *Agrobacterium* containing the corresponding construct using the leaf blade scratch method. Buds were then induced, and positive transgenic buds were transplanted into Murashige and Skoog (MS) media after hygromycin screening and PCR testing. The stable transformation lines (1, 10, and 12) were selected by qRT-PCR tests (S-Fig. 1a, b). Subculture and expanded propagation of lines 1, 10, and 12 were conducted for follow-up assays.

### Subcellular localization of McMYB4

We transformed the *pH7FWG2-McMYB4*-*GFP* vector into *Agrobacterium tumefaciens* GV3101. The *A. tumefaciens* populations were cultivated to saturation (OD_600_ = 1.0) in Luria-Bertani media, and the culture was then centrifuged. The thallus was resuspended in a solution of 10 mM MgCl_2_, 10 mM 2-(N-morpholino) ethanesulfonic acid (MES), and 150 mM acetosyringone. *Nicotiana benthamiana* was selected for four weeks and infected via the back of the leaves with a needle-free syringe. The leaves were cultivated for two days and then subjected to laser confocal microscopy (LEICA SP8) observation.

### Transcriptome analysis

We selected tissue culture seedlings of *McMYB4*-OE transgenic “Golden Delicious” apple (line 1) and “Golden Delicious” apple (WT) for transcriptome sequencing analysis and utilized three sampling times for each plant. The samples were ground immediately in liquid nitrogen, and total RNA was extracted using TRIzol reagent (Invitrogen) according to the manufacturer’s protocol. Determination of RNA quantity and purity, the addition of adapters, size selection, and RNA-seq were performed by Novogene (Beijing, China). An RNA-sequencing library was prepared and sequenced using the Illumina HiSeq^TM^ platform. Quality control (QC) was performed by analyzing the error rate distribution along reads and the classification of raw reads. DEGseq 1.12.0 was used to analyze the DEGs in each sample based on the criteria |log2(Fold change)|>1 and *q* < 0.005. Based on the DEG analysis, a KEGG analysis was conducted using KOBAS v2.0^52^.

### Measurement of the flavonol, lignin and hormone contents

HPLC was used to measure the flavonol content. Approximately 0.8–1.0 g (fresh weight) of each sample was placed in a test tube containing 10 mL of extract solution (methanol:water:formic acid:trifluoroacetic acid = 70:27:2:1). The samples were then shaken every 6 h during incubation at 4 °C in the dark for 72 h, and the samples were filtered and analyzed by HPLC^6^. The lignin content was measured as described previously and is expressed as OD_280_ μg·g^−1^ (DW)^34^. The contents of endogenous IAA/BR were detected by enzyme-linked immunoassay. The data are presented as the means ± SDs from three repeated experiments.

### Microscopic examination

After staining with DPBA (25 mg of DPBA dissolved in 10 mL of methyl alcohol) for 30 min, the leaves of tissue culture seedlings of WT and transgenic “Golden Delicious” apple were subjected to microscopy, and the samples were transferred to an objective table for color observations under UV light (Eclipse 80i fluorescence microscope). Cross-sections of the petioles of tissue culture seedlings of WT and transgenic “Golden Delicious” apple were observed under UV light, and images were collected and analyzed using digital imaging software.

### Y1H assays

The *McMYB4* CDS was cloned into a *pJG4-5* vector (Clontech) with the galactokinase 1 (GAL1) promoter, which served as an effector construct. The flavonol biosynthesis gene (*CHS/FLS*) promoter sequences were cloned into a *pLacZi* vector with the *LacZ* reporter gene. The vectors were transformed into competent cells of the yeast strain EGY48, which yielded the following yeast strains: *pJG4-5-McMYB4/pLacZi*-promoter of flavonol regulation genes and *pJG4-5/pLacZi*-promoter of flavonol regulation genes. The cells were selected on SD-Trp/-Ura media, and positive colonies were spotted onto glucose plates (2%) containing X-gal at 28 °C for two days to confirm the development of a blue color.

Simultaneously, the *McMYB4* CDS was also cloned into a *pGADT7* vector (*pGADT7-McMYB4*), the promoter sequences of the lignin biosynthesis genes *CAD* and *F5H* and hormone signal genes (*AUX/ARF/BRI/BIN*) were inserted into a *pAbAi* vector, and the mutant sequences were also inserted into a *pAbAi* vector as a negative control. Then, 200 ng of *pGADT7-McMYB4* was added to an Eppendorf (EP) tube, 50 μl of Y1H gold (*pCAD-Bait-AbAi*, *pF5H-Bait-AbAi*, *pAUX-Bait-AbAi*, *pAUX Mutant-AbAi*, *pARF-Bait-AbAi*, *pARF Mutant-AbAi*, *pBRI-Bait-AbAi*, *pBRI Mutant-AbAi*, *pBIN-Bait-AbAi*, and *pBIN Mutant-AbAi*) was added, and Y1H gold (*p53-AbAi*) was used as a positive control. Cells at different concentrations were selected on SD/-Ura, SD/-Leu, and SD/-Leu/500 ng AbA culture plates, and positive colonies grew normally^53^.

### EMSAs

The *McMYB4* CDS was cloned into a *pMAL-C2X* expression vector, and the resulting vector was subsequently transformed into *Escherichia coli* Rosetta (DE3) competent cells. The MBP tag was also cloned in the *pMAL-C2X* vector to purify the recombinant protein. Isopropyl β-D-1-thiogalactopyranoside (IPTG; 0.3 mM) was used to induce McMYB4 expression at 170 rpm for 6 h at 28 °C. The recombinant protein was purified using a One-Step MBP-Tagged Protein Mini-prep Pack (BioLab Co., Ltd., Beijing, China). EMSAs were conducted using the Light Shift^®^ Chemiluminescent EMSA Kit (Thermo Fisher Scientific) according to the manufacturer’s protocol with 10 μg of purified McMYB4 protein and *CHS*/*FLS*/*CAD*/*F5H* and *AUX*/*ARF*/*BRI*/*BIN* probes with biotin-labeled oligonucleotides^53^.

### ROS and enzyme activity assays

Leaf tissues (0.1 g) were ground to powder in liquid nitrogen and then added to an extraction solution. The O_2_^-^ and H_2_O_2_ contents and enzyme (APX, CAT, and SOD) activities were measured with kits (Solarbio) according to the manufacturer’s instructions. The following formulas were used for calculation: O_2_^-^ (nmol/g) = 148.76*(A_530 nm_ sample-A_530 nm_ control + 0.0027) / W, (W = 0.1 g); H_2_O_2_ (μmol/g) = ΔA_415 nm_ sample / ΔA_415 nm_ control / W (W = 0.1 g); APX (U/g) = 1.79*[(A3-A4)-(A1-A2)] / W, (control: A1 = A_290 nm_ 10 s, A2 = A_290 nm_ 130 s, sample: A3 = A_290 nm_ 10 s, A4 = A_290 nm_ 130 s, W = 0.1 g); CAT (U/g) = 678*(A1-A2)/W, (A1 = A_240 nm_ 5 s, A2 = A_240 nm_ 65 s, W = 0.1 g); and SOD (U/g) = 11.4*[(ΔA_560 nm_ control-ΔA_560 nm_ sample) / ΔA_560 nm_ control*100%] / [1-(ΔA_560 nm_ control-ΔA_560 nm_ sample) / ΔA_560 nm_ control*100%] / W*10 (30 min, W = 0.1 g).

### Data analysis

Statistical analyses and figure preparation were performed with OriginPro 8 (Origin Lab Corporation, USA) and Photoshop. The error bars for each symbol indicate the means ± SDs of three biological replicate reactions. The statistical analyses in the study were performed using Student’s *t* test, where * 0.01 < *P* < 0.05 and **P < 0.01.

## Supplementary information

Supplemental Figures

AJE certificate
