# Supplementary material for: McMYB4 improves temperature adaptation by regulating phenylpropanoid metabolism and hormone signaling in apple
Source: Hortic Res. 2021 Aug 1;8:182. doi: 10.1038/s41438-021-00620-0 (PMC8325679; doi:10.1038/s41438-021-00620-0)
Supplement: Supplementary file 1 — Supplemental Figures [file 41438_2021_620_MOESM1_ESM.doc]

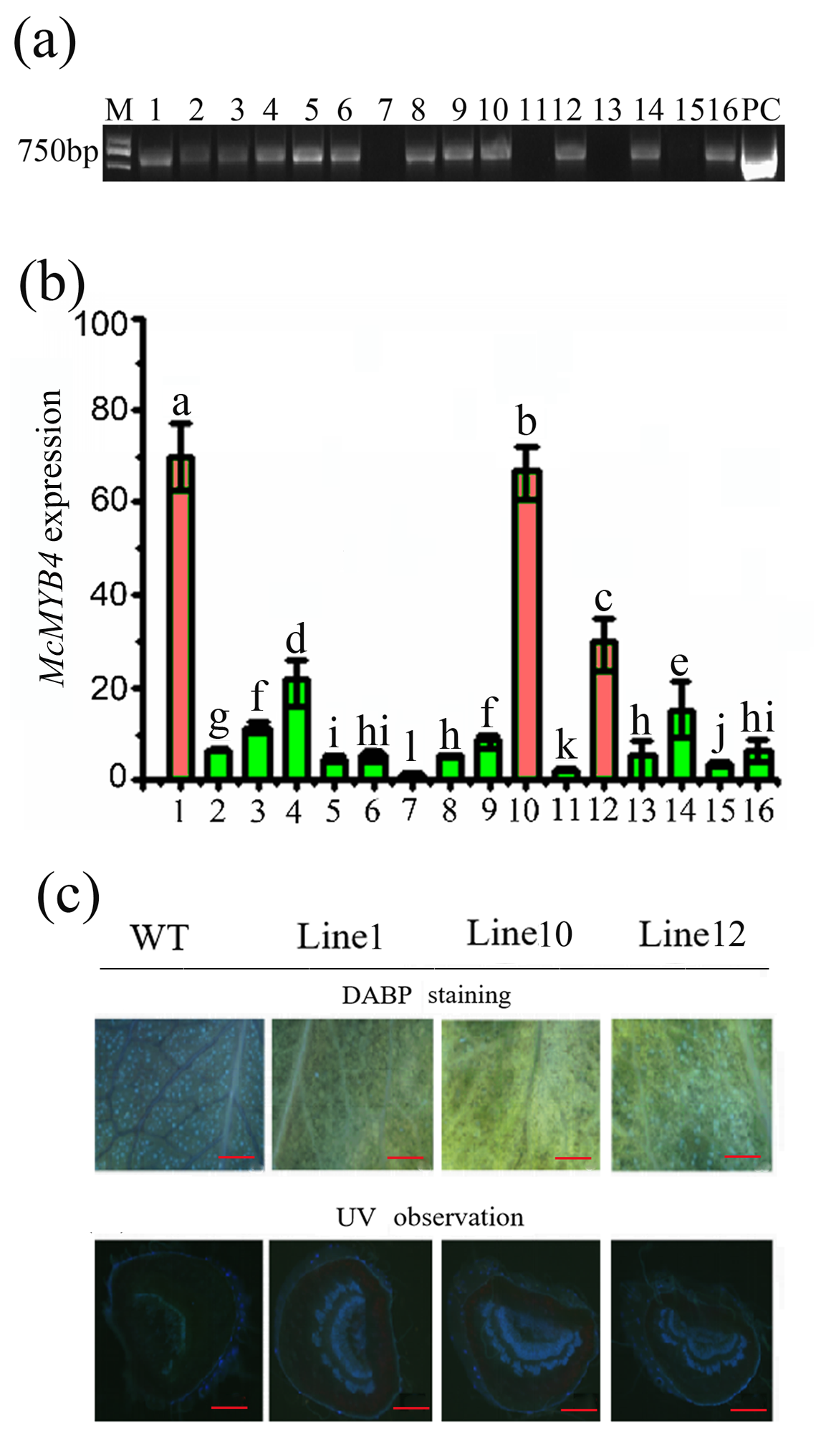


**Supplemental Figure 1** **Accumulation of flavonol and lignin in the WT and *McMYB4*-OE ‘Golden delicious’ apple lines. (a)** The electrophoretic bands were compared to the positive plasmid control (PC) by PCR using GFP primers. **(b)** Relative expression levels of *McMYB4* in different transgenic apple lines (L1-L16). Each bar indicates the mean±SD of three repeated experiments. **(c)** Flavonol accumulation in the blades of WT and transgenic lines (line 1, 10, and 12) according to DPBA staining. Bar=400 μm. Lignin accumulation in cross-sections of the petioles of leaves of the WT and transgenic lines (line 1, 10, and 12), as detected via an upright fluorescence microscope under UV light. Bar = 400 μm.


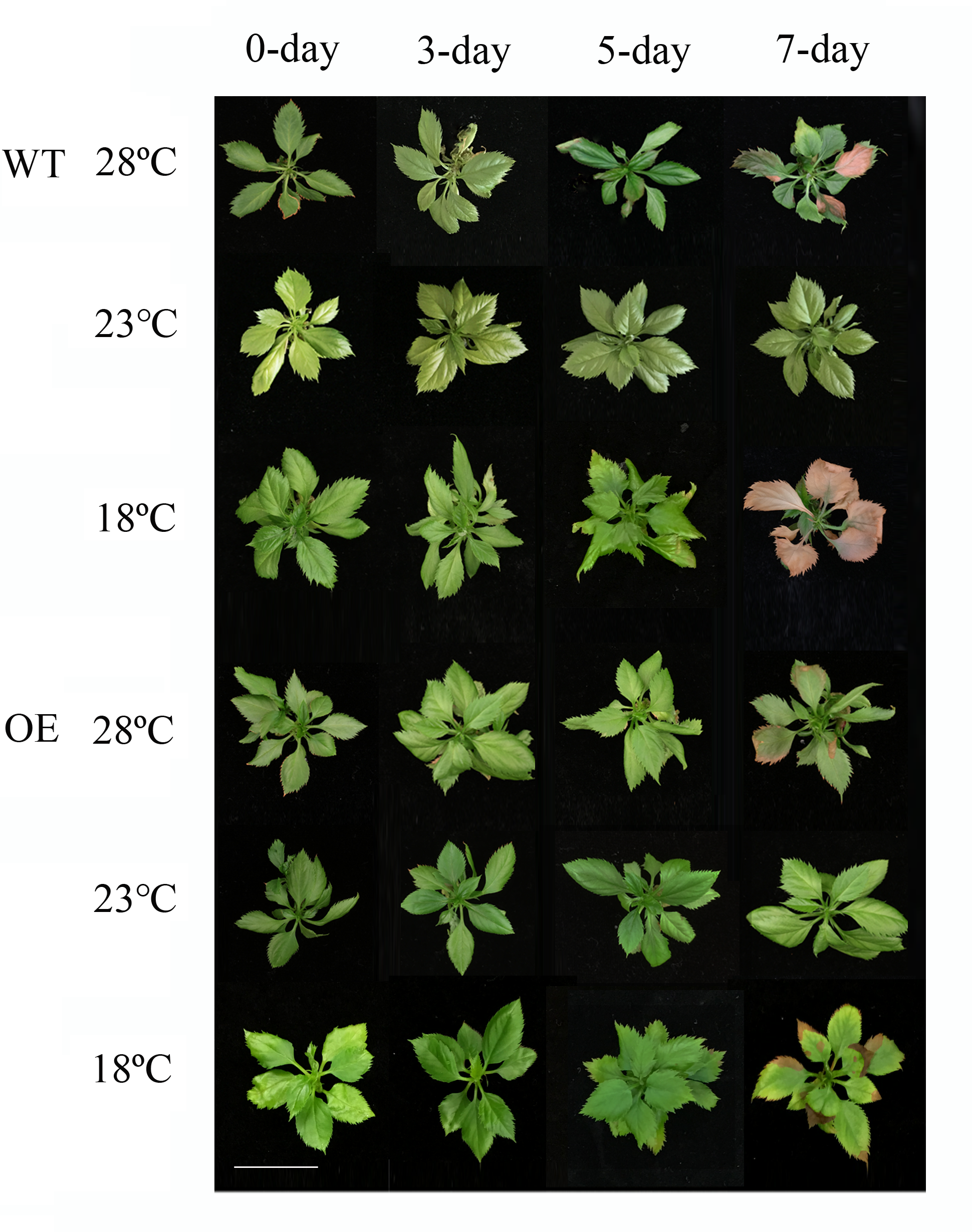


**Supplemental Figure 2 Phenotype of the WT and *McMYB4*-OE lines after treatment at 28ºC and 18ºC for 0, 3, 5, and 7 days (23ºC served as the control).** Bar = 2 cm.


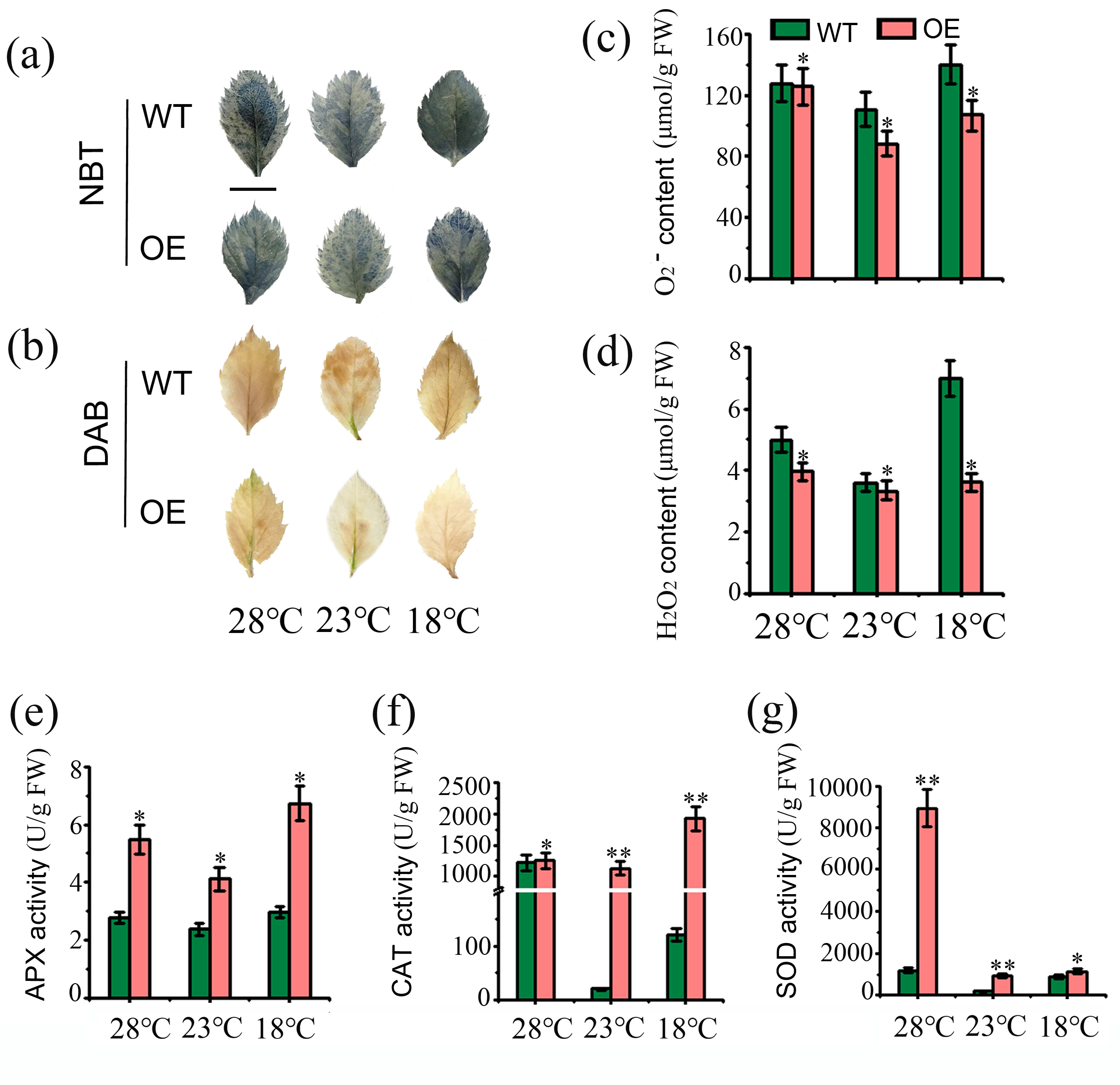


**Supplemental Figure 3** **ROS levels and enzyme activities in the WT and *McMYB4*-OE lines in response to temperature changes. (a and b)** Accumulation of O_2_^-^ and H_2_O_2_ in the WT and OE lines determined by nitro blue tetrazolium (NBT) (dark blue) and diaminobenzidine (DAB) (dark brown) staining respectively. The lines were subjected to the 28ºC and 18ºC treatments, and treatment at 23ºC served as the control. Bar=0.5 cm. **(c)** O_2_^-^ content in the leaves of WT and OE lines subjected to the 28ºC and 18ºC treatments; 23ºC served as the control. **(d)** H_2_O_2_ content in the leaves of the WT and OE lines grown at 28ºC and 18ºC; treatment at 23ºC served as the control. **(e-g)** Enzyme activities of APX, CAT, and SOD in the WT and OE lines under the 28ºC and 18ºC treatments; 23ºC served as the control. Each bar indicates the mean±SD of three repeated experiments (*P<0.05, **P<0.01, Student’s *t* test).
